# Genome-edited zebrafish model of *ABCC8* loss-of-function disease

**DOI:** 10.1080/19382014.2022.2149206

**Published:** 2022-12-02

**Authors:** Jennifer M. Ikle, Robert C. Tryon, Soma S. Singareddy, Nathaniel W. York, Maria S. Remedi, Colin G. Nichols

**Affiliations:** a Department of Cell Biology and Physiology, Washington University in St. Louis, St. Louis, Missouri, USA; b Center for the Investigation of Membrane Excitability Diseases, Washington University in St. Louis School of Medicine, St. Louis, Missouri, USA; c Department of Pediatrics, Washington University in St. Louis School of Medicine, St. Louis, Missouri, USA; d Department of Medicine, Division of Endocrinology, Metabolism, and Lipid Research, Washington University in St. Louis School of Medicine, St. Louis, Missouri, USA

**Keywords:** K_ATP_, metabolism, pancreas, zebrafish, calcium channels, insulin secretion

## Abstract

ATP-sensitive potassium channel (K_ATP_)gain- (GOF) and loss-of-function (LOF) mutations underlie human neonatal diabetes mellitus (NDM) and hyperinsulinism (HI), respectively. While transgenic mice expressing incomplete K_ATP_ LOF do reiterate mild hyperinsulinism, K_ATP_ knockout animals do not exhibit persistent hyperinsulinism. We have shown that islet excitability and glucose homeostasis are regulated by identical K_ATP_ channels in zebrafish. SUR1 truncation mutation (K499X) was introduced into *the abcc8* gene to explore the possibility of using zebrafish for modeling human HI. Patch-clamp analysis confirmed the complete absence of channel activity in β-cells from K499X (SUR1^−/−^) fish. No difference in random blood glucose was detected in heterozygous SUR1+/- fish nor in homozygous SUR1^−/−^ fish, mimicking findings in SUR1 knockout mice. Mutant fish did, however, demonstrate impaired glucose tolerance, similar to partial LOF mouse models. In paralleling features of mammalian diabetes and hyperinsulinism resulting from equivalent LOF mutations, these gene-edited animals provide valid zebrafish models of K_ATP_ -dependent pancreatic diseases.

## GRAPHICAL ABSTRACT

**Figure:**
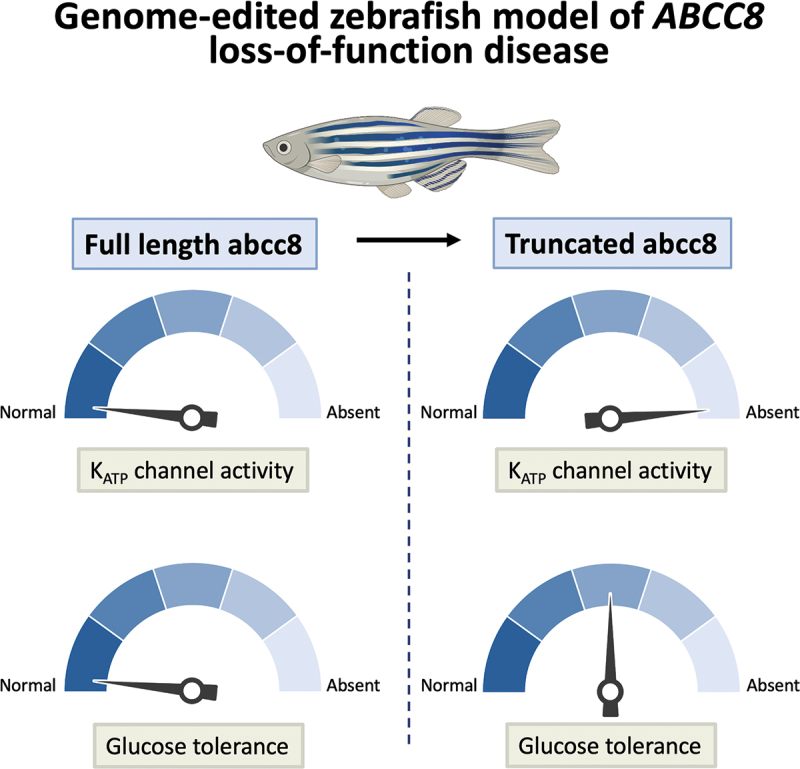

## Introduction

Electrical activity is a key regulator of insulin secretion from β-cells^1^ and is critically regulated by ATP-sensitive potassium (K_ATP_) channels. In mammals, pancreatic K_ATP_ channels are composed of four SUR1 subunits (encoded by *ABCC8*) and four Kir6.2 subunits (encoded by *KCNJ11*).^2,3^ At low plasma [glucose], K_ATP_ channels are normally open, the cell membrane is hyperpolarized, and voltage-dependent calcium channels (VDCCs) are closed, thus inhibiting insulin secretion.^4^ Glucose metabolism increases the intracellular [ATP]/[ADP] ratio via enhanced β-cell glycolysis and oxidative phosphorylation. This causes closure of the K_ATP_ channels, leading to membrane depolarization, calcium influx through VDCCs, and triggering of insulin release.^5,6^ Congenital hyperinsulinism (CHI) is the most common cause of hypoglycemia in neonates and infants^7^ and is often linked to loss-of-function (LOF) mutations in K_ATP_. LOF CHI mutations in either *KCNJ11* or *ABCC8*^8^ result in reduced K_ATP_ channel activity, β-cell hyperexcitability, and excessive insulin secretion.^9^ In direct contrast, gain-of-function (GOF) mutations in these same genes cause decreased membrane excitability and impaired insulin secretion, leading to neonatal diabetes mellitus (NDM).^6,10,11^ Mice with transgenic overexpression of GOF mutations first predicted a mechanism for human NDM and have provided valuable models for understanding disease progression.^12–14^ Similarly, mice with transgenic expression of K_ATP_ LOF mutations, as well as mice with heterozygous *KCNJ11* or *ABCC8* gene knockout, reiterate persistent hyperinsulinism.^15,16^ However, homozygous K_ATP_ knockout mice do not exhibit persistent hyperinsulinism; instead, they exhibit an unexplained loss of insulin secretion and glucose intolerance.^17–20^

Current therapeutic approaches to both NDM and CHI will benefit from novel animal models and new insights into disease processes, leading the way to new opportunities for treatment. We have shown that K_ATP_ channels are expressed in β-cells within the zebrafish (*Danio rerio*) islet, that they are functionally similar to their mammalian orthologues,^21^ and that they exert similar glucose-dependent control of intracellular [Ca2+] ([Ca2+]_i_).^22,23^ Activation of these channels by the drug diazoxide^21^ or by overexpression of ATP-insensitive transgenes in β-cells^23^ can similarly alter the metabolic response to glucose. To provide a model of K_ATP_ LOF, we have investigated a zebrafish model of the loss-of-function of K_ATP_ in which an early nonsense mutation, predicted to lead to premature truncation of SUR1, was introduced into *abcc8*. This zebrafish mutant recapitulates key features of human K_ATP_ LOF and provides a model for further analysis and testing of potential therapeutics, which may facilitate advances in clinical management and help identify new therapies by providing a high throughput platform for understanding mechanisms and testing potential therapeutic approaches.

## Materials and methods

### Ethical approval

All animal procedures were approved by the Washington University in St. Louis Institutional Animal Care and Use Committee.

### SUR1 ENU generated nonsense mutation

ENU-mutagenesis was performed at the Sanger Institute, as part of the Zebrafish Mutation Project, using N-ethyl-N-nitrosourea (ENU) mutagenesis to attempt to identify knockout alleles for all protein-coding regions in the zebrafish genome (https://www.sanger.ac.uk/resources/zebrafish/zmp/). This project outcrosses ENU-mutagenized F_0_ males to create a population of F_1_ fish heterozygous for ENU-induced mutations, which were then obtained through the Zebrafish International Research Consortium (ZIRC). The *abcc8(sa15863)* nonsense mutant allele (K499-STOP, TTCTGGCTCCRGTGCAGTACTTTGTGGCAACCAAGTTATCAGATGCACAG**[A > T]**AAAGCACATTGGTGAGCTACTTTATTTTGGTTAATGTCCTAATGAGGCCA) was obtained from the Zebrafish Mutation Project,^24^ through ZIRC. Homozygous K499-STOP mutants were generated by in-crossing heterozygous carriers, and the progeny was genotyped by Transnetyx using restriction digest with the inserted digestion site for HpyCHRIII, which is inserted into the mutant allele (Forward primer: TTGTTGTTGTCTGCTTTTTGC; Reverse primer: TTTACAAGCACAGCGCTCAC) to identify homozygotes.

### Animal lines and maintenance

In addition to the mutant lines above, we used AB wild-type fish as well as the previously described β-cell-specific GCaMP6s-expressing transgenic fish *Tg(−1.0ins:gCaMP6s)^stl44125^* and insulin reporter line *Tg(−1.0ins:eGFP)^sc1^*.^25^ Wild-type controls were on the AB background. All fish lines were housed in the Washington University Zebrafish Facility under standard conditions, the details of which can be found at: http://zebrafishfacility.wustl.edu/documents.html. Briefly, tanks and feeding are managed on the Tritone robotic system. Beginning day 4–6 post fertilization, larvae are housed at a density of 8–10/L and fed a combination of microalgae and/or rotifers. As the fish progress in their growth and development, larger food items will be provided, until they are moved to the adult fish-holding areas. If live foods are not available for first-feeding larvae, dry diets may be used. Adult fish are moved from the Nursery to the Adult Fish Holding rooms at ~42-days, or once 50% of the tank has reached sexual maturity, and are housed at a density of <1–12 fish/L. Adult fish held on the recirculating system are fed a minimum of once per day and may be fed up to five times per day, using prepared dry food and/or rotifers. Unless otherwise stated, all experiments were performed on adult zebrafish of reproductive age (10 weeks to 9 months of age) and on roughly equal proportions of males and females.

### Electrophysiological analyses

Islets were isolated from zebrafish, and single β-cells were dissociated, as previously described, and recordings were performed on GFP-positive cells.^21^ Excised patch recordings were performed using pipettes with a resistance of 1–2 MΩ when filled with pipette solution. Bath and pipette solution (K-INT) contained (in mM): 140 KCl, 10 HEPES, 1 EGTA (pH 7.4 with KOH). All recordings were performed at −50 mV holding potential, and the absence or presence of nucleotides was adjusted in bath solution as indicated. K_ATP_ currents were normalized to the basal current in the absence of nucleotides. The data were tested for statistical significance using Welch’s *t*-test. A *p* value of <0.05 was considered significant.

### Isolation of islets and β-cells

Islets and β-cells were obtained as described.^21^ Briefly, fish were euthanized using cold-shock (2–4°C water immersion) followed by decapitation. Under a fluorescent dissecting microscope, fish were placed onto their right sides, and the exterior skin and scales were removed using surgical forceps to expose the abdomen. Visceral organs were gently dissected away with forceps. The primary islets were identified at the intersection of hepatic and bile ducts with the intestine and confirmed by eGFP fluorescence. Islets were removed by gently pinching the ducts with forceps and separating the islets from the surrounding tissues and stored in islet media (see below) until all dissections were complete.

Islets were digested with collagenase (Sigma C9263, 0.4 mg ml^−1^ in Hank’s buffered salt solution, 0.5 ml/5–10 islets) to remove surrounding exocrine and connective tissues by incubation at 29°C for 20 min, shaking gently every 5 min. Islets were then placed in Islet Media made up of RPMI (ThermoFisher 11875–093) supplemented with 1 mM HEPES, antibiotic solution (Sigma A5955, 10 ml l^−1^solution), 10% fetal bovine serum and diluted with glucose-free RPMI to a final glucose concentration of 6.67 mM.

For experiments involving individual β-cells, islets were dispersed with StemPro Accutase (ThermoFisher A11105) for 10 min at 37°C. Any remaining clumps of cells were incubated a second time in the same conditions for 2 min. Dispersed cells were washed with islet media and re-suspended in less than or equal to 100 µl of media, then transferred to glass shards cut from coverslips. Cells were allowed to adhere for 30 min in incubator (28°C, 0% CO_2_) on shards before being completely covered with media and incubated overnight in the same conditions.

### Ex-vivo microscopy of adult zebrafish islet calcium

Islets were isolated and perfusion imaging experiments performed as described.^21^ Briefly, glass-bottomed 35 mm dishes (MatTeK) were coated with 1% agarose. A well was created in the center of the plate using a plastic pipette tip to remove a section of agarose. Islets were individually transferred to wells and immersed in pH 7.4 Krebs Ringer’s solution buffered with HEPES (KRBH) containing 2 mmol/L glucose. The KRBH base solution consisted of (in mmol/L): NaCl 114, KCl 4.7, MgSO_4_ 1.16, KH_2_PO_4_ 1.2, CaCl_2_ 2.5, NaHCO_3_ 5, and HEPES 20, with 0.1% BSA. KRBH solutions of indicated glucose concentrations were flowed into the plate chamber through lines running into and out of the chamber.

High-resolution images were captured using a Nikon Spinning Disk confocal microscope (a motorized Nikon Ti-E scope equipped with PerfectFocus, a Yokogawa CSU-X1 variable speed Nipkow spinning disk scan head, and Andor Zyla 4.2 Megapixel sCMOS camera) at the Washington University Center for Cellular Imaging (http://wucci.wustl.edu/). Time-lapse images used 100 msec exposure at 1 sec intervals. All images were analyzed in FIJI.^26^ To correct for movement in x- and y-planes, images were stack registered (using StackReg, rigid body) in Fiji before analysis. A single z-stack for each time-lapse was analyzed, with a region of interest (ROI) drawn to surround the border of the islet. Because the baseline electrical activity of an islet, and thus the intensity of islet fluorescence, can vary in our *abcc8* mutants, fluorescent response to glucose is shown normalized using min-max normalization.

### Quantification of islet β-cell density

To calculate β-cell density, whole islets were isolated as above and imaged in low glucose conditions at a single time point. A single representative z-stack was analyzed for each islet using FIJI software to calculate the islet area. Cells were counted using the FIJI plug-in StarDist.

### Blood glucose measurements and glucose tolerance test

Blood glucose was measured as described in random (fed) adult zebrafish.^21^ Zebrafish were fasted for 18–20 hours prior to glucose tolerance tests. Intraperitoneal glucose tolerance test was performed as described^21^ on similarly fasted zebrafish.

### Growth measurements

To obtain growth data, fish were briefly anesthetized in tricaine, and excess water removed with Kimwipe and then weighed on a digital scale with a precision of 1 µg, before returning the fish to reverse osmosis water to recover from anesthesia.

### Chemicals

All salts, amino acids, and other compounds were purchased from Sigma, except where indicated above.

### Statistics

Statistical analyses were performed in GraphPad Prism. Data on blood glucose over time and glucose tolerance test were tested for statistical significance using a one- or two-way ANOVA with Tukey test. Data on animal weight and β-cell density used Welch’s *t-*test and measurements of relative fluorescence used multiple *t*-tests. A *p* value of <0.05 was considered significant.

## Results

### Genome-modified zebrafish model of SUR1 LOF

**Figure 1.**
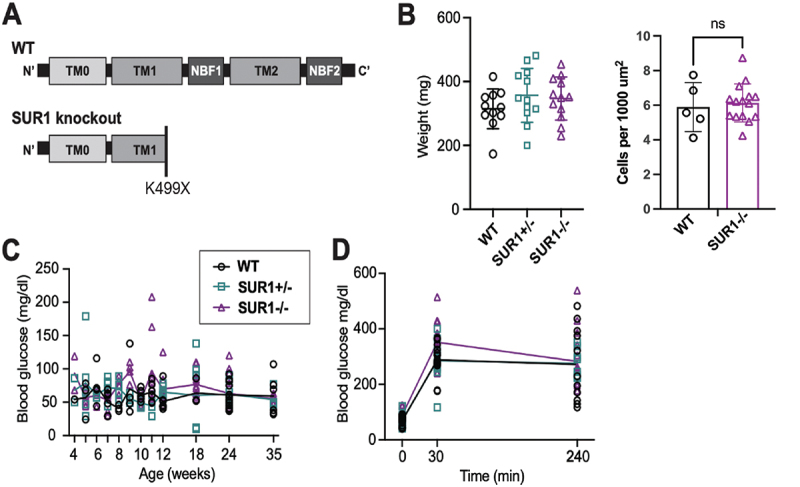
**Genome-modified zebrafish model of SUR1 LOF** (a) Schematic of SUR1 protein structure showing functional domains and indicating location of premature stop mutation (K499X) within TMD1. (b) Body weight in WT (n = 11), heterozygous SUR1 mutants (n = 12) and SUR1 knockout (n = 12) fish at age 8 weeks. Density of β-cell within individual islets is not different between WT (n = 5) and SUR1 mutant (n = 15). (c) Fasting blood glucose over time shows no significant difference between homozygous mutants and controls (n = 11–16 per timepoint). (d) Blood glucose versus time in response to glucose load in SUR1^−/−^, WT and SUR1+/- fish (n = 8–16 per timepoint).

A number of K_ATP_ mutations have been described to cause CHI, with the causal mutation being in the SUR1 subunit more often than in Kir6.2.^9,27^ Although gating mutations are a common underlying cause, mutations that result in loss of functional protein or failure to traffic to the cell membrane are also prominent.^5,28^ To model loss of functional protein, we obtained an *abcc8* mutant line that was originally generated by ENU mutagenesis, from the Zebrafish Mutation Project.^24^ These fish contain an early stop codon (X) mutation in exon 10 of SUR1 (K499X), which results in disruption of transmembrane domain 1 (Figure 1a), and is expected to result in complete loss of functional SUR1 protein. Complete knockout of channel activity was confirmed in isolated membrane patches of β-cells from homozygous mutant fish (see below), which we thus term SUR1^−/−^. Infants with CHI often have macrosomia,^29,30^ but, as with mouse SUR1 knockouts,^18,19,31^ there was no significant difference in growth between SUR1-/- mutants and wild type (Figure 1b). Islet development, as indicated by β-cell density, also showed no difference in the mutants (Figure 1b). In mice with such a marked loss of K_ATP_ channels, a common theme of early transient hyperinsulinemic hypoglycemia followed by normoglycemia or impaired glucose tolerance as adults has been described.^16,18,19,31^ A similar progression has also been seen in some humans with CHI due to K_ATP_ LOF.^28,32,33^ Preliminary measurements suggest that SUR1^−/−^ larvae also have lower whole-body glucose compared to wildtype (not shown), but random fed blood glucose in both homo- and heterozygous fish was not different from WT (Figure 1c).

Intraperitoneal glucose tolerance test (IPGTT) was performed on adult zebrafish. We have previously shown that IPGTT in wild-type zebrafish has a peak in blood glucose around 30 minutes with a slow return to normal over four to six hours.^21^ Here, we found higher baseline fasting and higher peak glucose in SUR1^−/−^ compared to wild type, but similar glucose values by 4 hours post injection (Figure 1d). This parallels the findings in mouse SUR1 and Kir6.2 knockout models of complete loss of K_ATP_, which lack persistent hypoglycemia and instead exhibit glucose intolerance and loss of insulin secretion as adults.^17–20^

### Molecular consequences of introduced gene modifications

**Figure 2.**
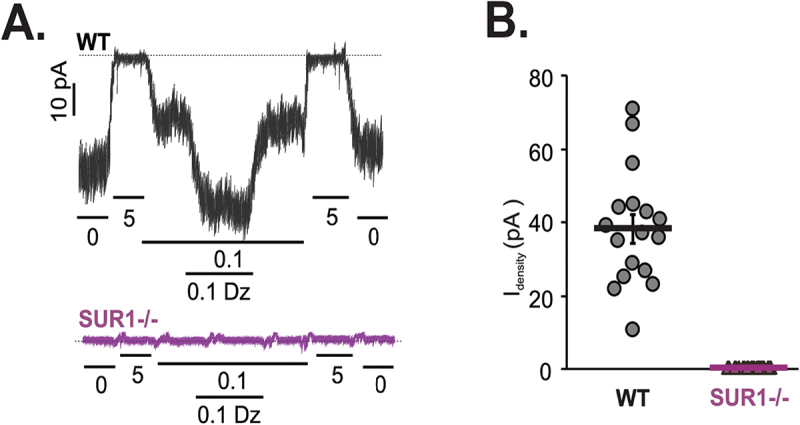
**SUR1 LOF [499X] abolishes K_ATP_ channel activity** (a) Representative K_ATP_ channel activity in inside-out patch clamp recordings from inside-out patch clamp recordings of WT (above) and homozygous SUR1[499X] (SUR1^−/−^) mutant β-cells (below) in the presence of ATP (μM), or addition of diazoxide (mM), as indicated. Voltage was clamped at −50 mV. (b) KATP channel density in WT and SUR1-/- patches (n = 17,10).

We crossed SUR1 mutant fish to *ins:GFP* expressing fish to assess morphology and insulin gene promoter activity. GFP fluorescence was used to identify β-cells in isolated islets via confocal microscopy. There was no obvious deficit of β-cell density or GFP density in SUR1 mutants compared to WT (not shown). Inside-out patch clamp recordings from β-cells isolated from primary islets of SUR1 homozygous mutant and wild-type fish, both containing *ins:GFP* transgene, were also identified by the presence of green fluorescence. Excised inside-out patch-clamp experiments (Figure 2a,b) confirmed the effective knockout, with complete absence of K_ATP_ channels in SUR1 homozygous mutant β-cells. These recordings additionally confirmed no responsivity to the channel opener diazoxide, consistent with a severe loss of function.

### Excitability consequences of introduced gene modifications

**Figure 3.**
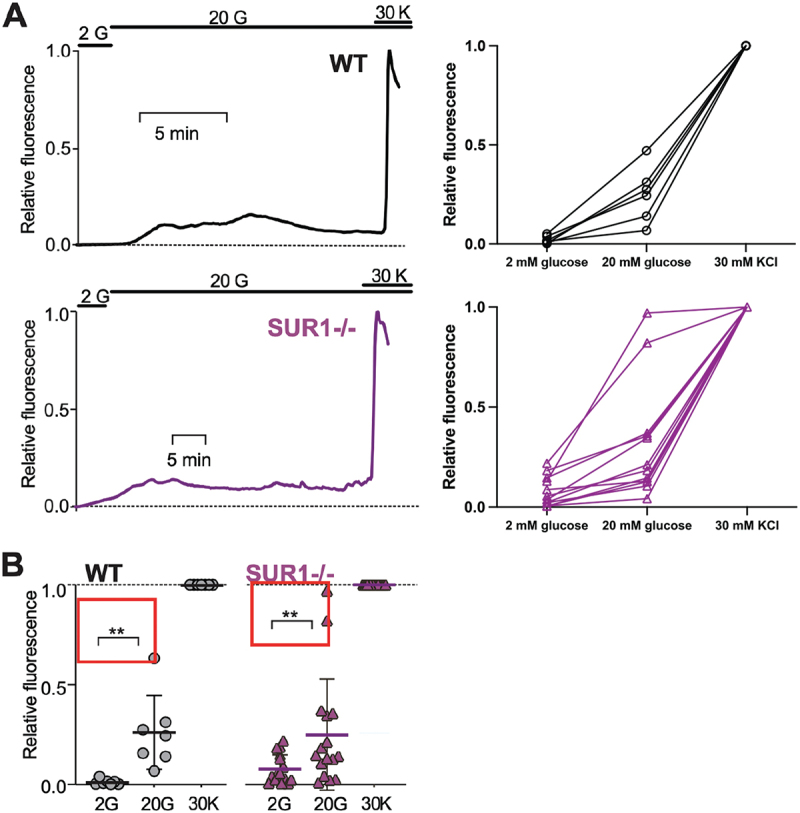
**SUR1^−/−^ islets exhibit elevated basal [Ca2+] and reduced responsivity to glucose** (a) Representative recordings of intracellular calcium in the presence of 2 mM glucose (2 G) and following switch to 20 mM glucose (20 G) and then 30 mM KCl (30 K), for WT (*above*, n = 6) and SUR1^−/−^ (*below*, n = 12). Fluorescence is normalized to maximum fluorescence in 30 K (*f* = 1), and minimum fluorescence (*f* = 0) anywhere within the record. Values for each islet are also shown. (b) Average calcium in each condition for WT (N = 8) and SUR1^−/−^ (n = 14). Data in B are analyzed by 1-way ANOVA followed by multiple unpaired t-tests. (*) p < .05, (**) p < .01.

K_ATP_ channel LOF is predicted to increase islet excitability and increase Ca2+ entry into β-cells. Mutant fish were crossed with transgenic GCaMP6s fish carrying *Tg(ins:GCaMP6s)*,^23^ and time-lapse confocal fluorescent microscopy was carried out on *ex vivo* perifused whole adult islets. Consistent with prior findings, controls showed a significant increase in relative fluorescence when transitioned from low (2 mM) to high (20 mM) glucose (Figure 3a, b). In SUR1 mutant zebrafish, carrying the same *Tg(ins:GCaMP6s)*, [Ca2+] imaging revealed elevated basal fluorescence at low (2 mM) glucose concentration, reflective of basal depolarization in mutant β-cells, but a smaller increase from baseline in high (20 mM) glucose, when compared to the increase seen in controls (Figure 3a, b). These results are very similar to previous findings in SUR1 knockout mice.^34^

## Discussion

In the current study, we have validated the first K_ATP_-knockout zebrafish and demonstrate a recapitulation of the essential consequences seen in mammalian K_ATP_ knockout animals. The premature stop mutation at position 499 is expected to result in a severely truncated protein, containing only the TMD0 region and half of the second TMD1 region and lacking both nucleotide binding folds (NBFs). Previous studies have shown that essentially no functional channels are formed when NBF1 is absent^35^ and, accordingly, we first show that homozygous ENU-generated SUR1 truncation mutant fish generate a functional K_ATP_ channel knockout, with no measurable K_ATP_ channels in isolated pancreatic β-cells. Secondly, this knockout results in elevated intracellular [Ca2+] at physiologically basal glucose levels (2 mM), even though [Ca2+]i is elevated at higher (20 mM) glucose, as is also seen in rodent SUR1 knockout islets.^34^

Thirdly, these fish reiterate the counterintuitive elevation of basal [glucose] and relative glucose intolerance that is seen in mammalian K_ATP_ knockout animals.^36^ It is likely that many human HI mutations will cause only incomplete loss of K_ATP_ channel activity,^37,38^ and both active K_ATP_ channels and sensitivity to the K_ATP_ channel opener diazoxide have been detected in some HI patients with K_ATP_channel mutations.^28,39^ Mice with *partial* loss of K_ATP_ activity mimic this hyperinsulinemic phenotype and secrete insulin at lower [glucose] than controls.^16^ However, while mice with *complete* loss of K_ATP_ exhibit elevated serum insulin and hypoglycemia in the neonatal period,^16,18,19,31^ they then rapidly develop hyperglycemia with reduced insulin secretion, a phenomenon that persists through adulthood. This cross-over to loss of secretion, in the face of continual excitation, reflects a marked down-regulation of the secretory process itself. The underlying cause remains elusive, and it is unknown whether this is a mouse-specific progression or reflects processes that may also be involved in human HI, although the correlative data is substantial.^36^ In demonstrating a similar glucose-intolerant and non-hypoglycemic phenotype in zebrafish that completely lacks K_ATP_ channels, these SUR1^−/−^ fish thus confirm a common finding from fish to mouse and may provide a useful model for further exploring the unexplained phenomenon of glucose intolerance and even diabetes in K_ATP_-dependent HI patients.

## Conclusions

In paralleling features of mammalian hyperinsulinism resulting from equivalent loss-of-function mutation, these gene-edited animals provide a valid zebrafish model of K_ATP_ LOF dependent pancreatic diseases.

## Abbreviations

KCNJ11: potassium voltage-gated channel subfamily J member 11
ABCC8: ATP Binding Cassette Subfamily C Member 8
Kir6.2: ATP-sensitive potassium channel pore subunit, inward-rectifying
SUR1: Sulfonylurea receptor 1
VDCC: Voltage-dependent calcium channel
NDM: Neonatal diabetes mellitus

## Key points

- Gain and loss-of-function in the Kir6.2 (KCNJ11) and SUR1 (*ABCC8*)-encoded pancreatic islet β-cell K_ATP_ channel underlies neonatal diabetes mellitus (NDM) and congenital hyperinsulinism, respectively. Mouse models reiterate key features, but zebrafish models could provide a powerful model for further analysis and therapy testing.
- An early nonsense mutation in exon 10 of SUR1 was generated by ENU mutagenesis.
- Homozygous SUR1 truncation mutants had normal random glucose but impaired fasting glucose and glucose tolerance as adults, mimicking findings in mouse SUR1 knockouts.
- Patch-clamp analysis revealed an absence of KATP channels in the SUR1 truncation mutants.
- Ca2+ imaging demonstrated elevated basal [Ca2+]_i_ in β-cells with SUR1 truncation.
- In paralleling features of mammalian diabetes and hyperinsulinism resulting from equivalent loss-of-function mutations, this gene-edited animal provides a valid zebrafish model of K_ATP_ -LOF dependent pancreatic diseases.
